# Scientific American

**Published:** 1894-04

**Authors:** 


SCIENTIFIC AMERICAN. Architects' and Builders' Edition. Published monthly by Munn & Co., 361 Broadway, New

York. Subscription price, $2.50 per year. Single copies 25 cents.

The March number of this elegant periodical is issued and is very interesting with colored plates of fine houses and cottages, with full plans and copious descriptions. Many readers of THE HOMŒOPATHIC PHYSICIAN have from time to time incidentally mentioned in their correspondence with the editor of this journal that they are "building."

To all such we cordially recommend the publication under notice as a ready means of finding an architectural device that shall relieve their homes of the plainness and sameness that has been characteristic of dwellings in the past.

The Architects' and Builders' edition does not confine itself exclusively to great buildings as one might expect, but deals with cottages of all classes, even the humblest, making a specialty of teaching all who contemplate building unpretentious homes how to make them pleasing to the eye.

